# 204 The C.A.R.E. Framework: A Strategic Approach to Controlling Candida auris Transmission at an Acute Care Hospital in New York City

**DOI:** 10.1017/ash.2026.10591

**Published:** 2026-06-23

**Authors:** Cecilia Li, Tiffany Wu, Carissa Tedeschi, Alex Rock, Mei Li, Sarah Turbett

**Affiliations:** 1 Massachusetts General Hospital

## Abstract

**Background:** Stenotrophomonas maltophilia isolated from urine cultures often reflects colonization, however true urinary tract infection (UTI) can occur. At Massachusetts General Hospital, antimicrobial susceptibility testing (AST) is routinely performed on urinary S. maltophilia isolates when present in at least moderate amounts or is the predominating organism from a non-sterile culture source. We evaluated patients with S. maltophilia urinary isolates to characterize treatment practices, determine the proportion of isolates representing definitive UTI, and assess recurrence by treatment status to evaluate whether limiting routine AST could reduce laboratory workload and avoid unnecessary antibiotic prescribing. **Methods:** We performed a retrospective cohort study of patients with positive urine cultures containing S. maltophilia between May 1, 2021 and May 1, 2024. Patients were excluded if they were treated for asymptomatic bacteriuria due to pregnancy or an upcoming urologic procedure, had a repeat urine S. maltophilia isolate within 14 days of index culture, had concomitant systemic infection treated with an antibiotic active against S. maltophilia, or died before recurrence evaluation. The primary objective was to characterize treatment decisions when S. maltophilia was isolated in urine culture. Secondary objectives included determining the frequency of true S. maltophilia UTI and evaluating recurrence based on whether the infection was treated. We defined definitive UTI as localized urinary symptoms with pyuria; possible UTI as systemic symptoms not explained by other causes or localized symptoms with an alternative etiology, with pyuria; and unlikely UTI as no symptoms reported or documented with or without pyuria. **Results:** A total of S. maltophilia directed therapy, despite only 19 (15.6%) meeting criteria for definitive UTI. In 11 cases, treatment was administered despite UTI being classified as unlikely. Among total untreated urine cultures (n=83) and definitive infections that were untreated (n=7), recurrent S. maltophilia infection was uncommon (3.6% and 0%, respectively). **Conclusion:** In our cohort, few urinary S. maltophilia isolates were reflective of definitive UTI, and withholding targeted therapy was associated with low recurrence. These findings suggest many isolates reflect colonization, particularly in patients with urinary instrumentation, and support consideration of a more selective approach to AST and antibiotic prescribing when isolated in urine culture.

